# The Effect of CAG Repeats within the Non-Pathological Range in the HTT Gene on Cognitive Functions in Patients with Subjective Cognitive Decline and Mild Cognitive Impairment

**DOI:** 10.3390/diagnostics11061051

**Published:** 2021-06-07

**Authors:** Valentina Bessi, Salvatore Mazzeo, Silvia Bagnoli, Giulia Giacomucci, Assunta Ingannato, Camilla Ferrari, Sonia Padiglioni, Virginia Franchi, Sandro Sorbi, Benedetta Nacmias

**Affiliations:** 1 Department of Neuroscience, Psychology, Drug Research and Child Health, University of Florence, 50139 Florence, Italy; salvatore.mazzeo@unifi.it (S.M.); silvia.bagnoli@unifi.it (S.B.); giuliagiacomucci.md@gmail.com (G.G.); ingannato.assunta@gmail.com (A.I.); camilla.ferrari@unifi.it (C.F.); virginia.franchi1@stud.unifi.it (V.F.); sandro.sorbi@unifi.it (S.S.); benedetta.nacmias@unifi.it (B.N.); 2 IRCCS Fondazione Don Carlo Gnocchi, 50143 Florence, Italy; 3 Regional Referral Centre for Relational Criticalities, 50139 Tuscany Region, Italy; sonia_padiglioni@libero.it; 4 Unit Clinic of Organizations Careggi University Hospital, 50139 Florence, Italy

**Keywords:** subjective cognitive decline, mild cognitive impairment, Huntington’s gene, CAG repeats, intermediate alleles, cognitive functions, APOE, BDNF

## Abstract

The Huntingtin gene (HTT) is within a class of genes containing a key region of CAG repeats. When expanded beyond 39 repeats, Huntington disease (HD) develops. Individuals with less than 35 repeats are not associated with HD. Increasing evidence has suggested that CAG repeats play a role in modulating brain development and brain function. However, very few studies have investigated the effect of CAG repeats in the non-pathological range on cognitive performances in non-demented individuals. In this study, we aimed to test how CAG repeats’ length influences neuropsychological scores in patients with subjective cognitive decline (SCD) and mild cognitive impairment (MCI). We included 75 patients (46 SCD and 29 MCI). All patients underwent an extensive neuropsychological battery and analysis of HTT alleles to quantify the number of CAG repeats. Results: CAG repeat number was positively correlated with scores of tests assessing for executive function, visual–spatial ability, and memory in SCD patients, while in MCI patients, it was inversely correlated with scores of visual–spatial ability and premorbid intelligence. When we performed a multiple regression analysis, we found that these relationships still remained, also when adjusting for possible confounding factors. Interestingly, logarithmic models better described the associations between CAG repeats and neuropsychological scores. CAG repeats in the HTT gene within the non-pathological range influenced neuropsychological performances depending on global cognitive status. The logarithmic model suggested that the positive effect of CAG repeats in SCD patients decreases as the number of repeats grows.

## 1. Introduction

Subjective cognitive decline (SCD) is the self-experienced decline in cognitive capacity with normal performance on standardized cognitive tests [1]. Individuals with SCD are twice as likely to develop dementia as individuals without [2]. Mild cognitive impairment (MCI) describes subjects with objective cognitive impairment without an impact on instrumental activities of daily living [3], and it is considered a transitional state between the normal cognitive state and dementia. A growing number of studies have shown that the onset and neuropsychological features of SCD and MCI are influenced by demographic and genetic factors [4,5,6,7].

Huntingtin is a soluble peptide, which is widely expressed during development, being essential for embryogenesis [8], and it plays crucial roles in axonal trafficking, regulation of gene transcription, and cell survival in post-developmental life [9]. In particular, the Huntingtin protein specifically enhances the vesicular transport of brain-derived natriuretic factor (BDNF) [10], a neurotrophic factor involved in synaptic connections [11], neural growth [12], and synaptic plasticity [13].

The Huntingtin gene (HTT) contains a key region of simple sequence repeats (CAG), which is translated into a corresponding polyglutamine stretch [14]. The expansion of the CAG triplet leads to dysfunction and death of neurons in the striatum and in other brain regions, causing Huntington’s disease (HD) [15]. International guidelines for HD genetic testing define the CAG repeat range of 40 or more to be consistent with HD [16]. Individuals with 36 to 39 repeats are currently categorized as carriers of low-penetrance HHT repeat expansions and may show either a normal or an HD phenotype. Within the normal length range, CAG expansions ranging from 27 to 35 CAG are termed as intermediate alleles (IAs). IAs are considered not to be associated with HD, but they are unstable and prone to increasing their length to a pathological range in offspring [17]. The biological function of CAG repeat expansion below the non-pathological threshold is an interesting research topic for its potential ontogenetic and phylogenetic roles. Increasing evidence has suggested that simple sequence repeats play a substantial role in evolution [18], as well as in organogenesis [19,20], by providing the variability needed to enhance changes of brain development [21]. A higher number of repeats in HTT, below the disease threshold, confers advantageous changes in brain structure and general intelligence [22]. On the other hand, subjects carrying IAs experienced more depressive symptoms compared to control subjects [23,24,25]. Other studies reported a significantly higher frequency of HTT IAs in AD patients [26] and in the non-fluent variant of primary progressive aphasia [27], suggesting a role of the HTT gene in the pathogenesis of these diseases. Whether HTT CAG repeats also influence cognition in preclinical and in prodromic phases of AD has not been explored.

In the present study we aimed to assess the effect of CAG repeats in the HTT gene below the pathological threshold in a sample of patients with SCD and MCI.

## 2. Materials and Methods

### 2.1. Participants and Clinical Assessment

As part of a longitudinal, clinical–neuropsychological–genetic survey on SCD and MCI, we included 75 consecutive spontaneous patients who self-referred to the Centre for Alzheimer’s disease and Adult Cognitive Disorders of the Careggi Hospital in Florence. All the patients were Caucasian. Inclusion and exclusion criteria were described in a previous work by our group [6]. All participants underwent an extensive neuropsychological battery, assessment of cognitive complaints, and peripheral blood collection to analyze Apolipoprotein E (APOE), HTT, and BDNF genotypes.

We divided our sample into two groups: 46 patients classified as SCD, according to the terminology proposed by the Subjective Cognitive Decline Initiative (SCD-I) Working Group (i.e., presence of a self-experienced persistent decline in cognitive capacities with normal performance on standardized cognitive tests) [1]; 29 patients classified as MCI, according to the (NIA-AA) criteria for the diagnosis of MCI [3].

The local ethics committee approved the protocol of the study. All participants gave written informed consent. All procedures involving experiments on human subjects were done in accordance with the ethical standards of the Committee on Human Experimentation of the institution in which the experiments were done or in accordance with the Helsinki Declaration of 1975. Specific national laws have been observed.

### 2.2. Neuropsychological Assessment

All subjects were evaluated by means of an extensive neuropsychological battery [28]. The battery consisted of global measurements (Mini-Mental State Examination), tasks exploring verbal and spatial short-term memory (Digit Span; Corsi Tapping Test) and verbal long-term memory (Five Words and Paired Words Acquisition; Recall after 10 min; Recall after 24 h; Babcock Short Story Immediate and Delayed Recall), and language (Token Test; Category Fluency Task) [28]. Visual–spatial abilities were also evaluated by the Rey–Osterrieth Complex Figure copy (ROCF-C), and visual-spatial long-term memory was assessed by recall of the Rey–Osterrieth Complex Figure test (ROCF-R) [29]; attention/executive function was explored by the Dual Task [30], Phonemic Fluency Test [31], and the Trail Making Test part A (TMT-A), part B (TMT-B), and B-A (TMT B-A) [32]. Everyday memory was assessed by the Rivermead Behavioral Memory Test (RBMT) [33]. All raw test scores were adjusted for age, education, and gender according to the correction factor reported in validation studies for the Italian population [28,29,30,31,32,33]. In order to estimate premorbid intelligence, all cases were assessed at baseline by the Test di Intelligenza Breve (TIB, i.e., Brief Intelligence Test) [34], an Italian version of the National Adult Reading Test (NART) [35]. The presence and severity of depressive symptoms was evaluated by the 22-item Hamilton Depression Rating Scale (HRSD) [36]. Cognitive complaints were explored at baseline using a survey based on the Memory Assessment Clinics-Questionnaire (MAC-Q) [37].

### 2.3. HTT, BDNF and APOE Genotyping

Subjects’ DNA was isolated from peripheral blood using standard automated method (QIAcube, QIAGEN, Hilden, Germany). APOE genotypes were investigated by High Resolution melting Analyses (HRMA) [38]. Two sets of PCR primers were designed to amplify APOE regions, encompassing rs7412 [NC_000019.9:g.45412079C > T] and rs429358 (NC_000019.9:g.45411941T > C). The APOE genotype was coded as APOE ε4- (no APOE ε4 alleles) and APOE ε4+ (presence of one or two APOE ε4 alleles).

Analysis of BDNF rs6265 (Val66Met) polymorphism was performed using High HRMA. PCR primers were designed as follows: 5′-ACTCTGGAGAGCGTGAATGG-3′and 5′ ACTACTGAGCATCACCCTGGA-3′; the samples with known BDNF genotypes, which had been validated by DNA sequencing (310 ABI PRISM Genetic Analyzer, Applied Biosystem, Foster City, CA, USA), were used as standard references.

HTT CAG repeat expansion was determined by a polymerase chain reaction amplification assay, using fluorescently labeled primers [39]. The size of the fragment was determined by capillary electrophoresis using SeqStudio Genetic Analyzer (ThermoFisher, Waltham, MA, USA) and the GeneMapper version 4.0 software (Applied Biosystems, Foster City, CA, USA). A set of HTT CAG alleles, whose lengths were confirmed by DNA sequencing, was used to provide size standards.

### 2.4. Statistical Analysis

Scores of cognitive tests were reported as *z*-scores, calculated on the mean and standard deviation (SD) of the Italian general population, reported in literature for each neuropsychological test. We tested for normality by the Shapiro–Wilk test. Patient groups were characterized by using means and standard deviations, median and interquartile range (IQR), frequencies or percentages, and 95% confidence interval (95% CI) for continuous distributed variables, continuous non-normally distributed variables, and categorical variables, respectively. Depending on the distribution of our data, we used a t-test or non-parametric Mann–Whitney-*U* Tests for between-groups comparisons, Pearson’s correlation coefficient or non-parametric Spearman’s ρ (rho) to evaluate correlations between groups’ numeric measures, and chi-square tests to compare categorical data. We used multiple linear regressions for multivariate analysis. Analysis was done first using the allele with the longest repeat and then repeated using the shorter allele. All statistical analyses were performed with SPSS software v.25 (SPSS Inc., Chicago, IL, USA) and R 4.0.3 (R Foundation for Statistical Computing, Vienna, Austria, 2013).

## 3. Results

### 3.1. Frequency Distribution of CAG Repeats

**Figure 1:**
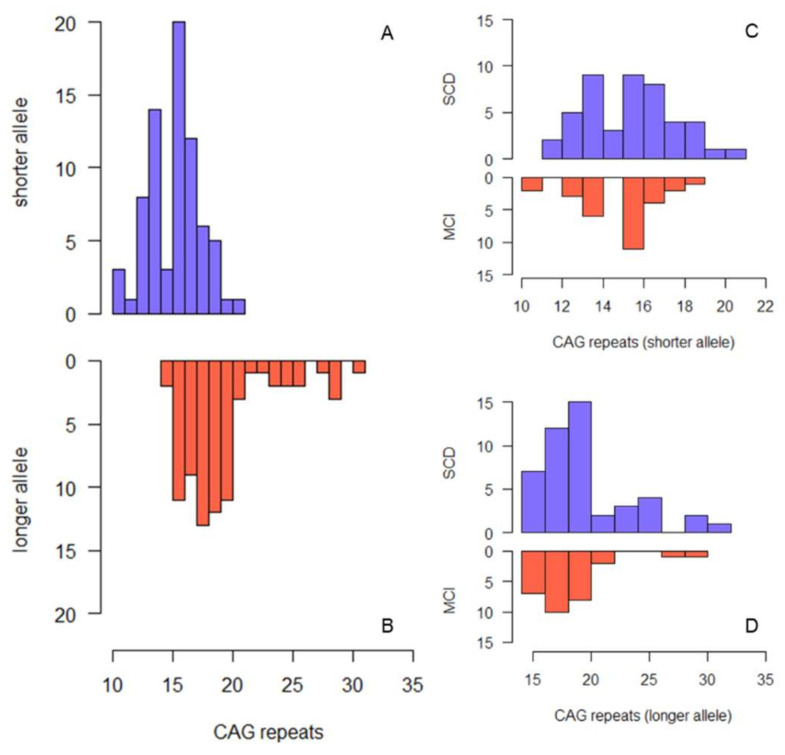
Histogram describing the frequency of CAG repeat lengths in the whole sample ((**A**) shorter allele, (**B**) longer allele) and in SCD and MCI separately ((**C**) shorter allele, (**D**) longer allele).

Figure 1A shows the frequency distribution of CAG repeats in the shorter and longer allele, respectively. The median CAG repeat length was 16.00 (IQR 3.00, range: 10–21) in the shorter allele and 19.00 (3.00, range: 14–31) in the longer allele. The most common HTT alleles had 16 (shorter alleles) and 18 (longer alleles) CAG-repeats. Five out of 75 patients (6.67%; 95% CI = 1.02:12.32) were heterozygous carriers of intermediate alleles of the HTT gene (IAs^+^). None of the patients were homozygous for intermediate alleles. In the whole sample, there were no differences between IAs^+^ and IAs^−^ with respect to all the considered demographic variables, the proportion of the APOE ε4 allele, MMSE, HDRS, and MAC-Q. None of the patients were taking anticholinergic drugs or memantine.

### 3.2. Comparison between SCD and MCI

SCD patients were younger at onset (56.00 (IQR 15.00) vs. 66.00 (IQR 13.00) years) and at baseline (61.52 (IQR 14.13) vs. 68.52 (IQR 13.44) years) and had more years of education (11.00 (IQR 8.00) vs. 8.00 (8.00) years) than MCI patients (Table 1). There were no differences between SCD and MCI with respect to the other considered variables. Figure 1C,D show the frequency distribution of CAG repeats in SCD and MCI, respectively. The median number of repeats in the longer HTT allele was slightly but significantly higher in SCD compared to MCI (19 (IQR 3.00) vs. 18 (IQR 2.50), *p* = 0.042). Three out of 46 SCD patients (6.52% (95% CI = 0:13.66)) and two out of 29 MCI patients (6.90% (95% CI = 0:16.12)) were heterozygous carriers of the intermediate allele of the HTT gene (IAs^+^). There was no difference in the proportion of intermediate alleles between SCD and MCI groups.

### 3.3. Correlations between Neuropsychological Scores and CAG Repeat Length

**Figure 2:**
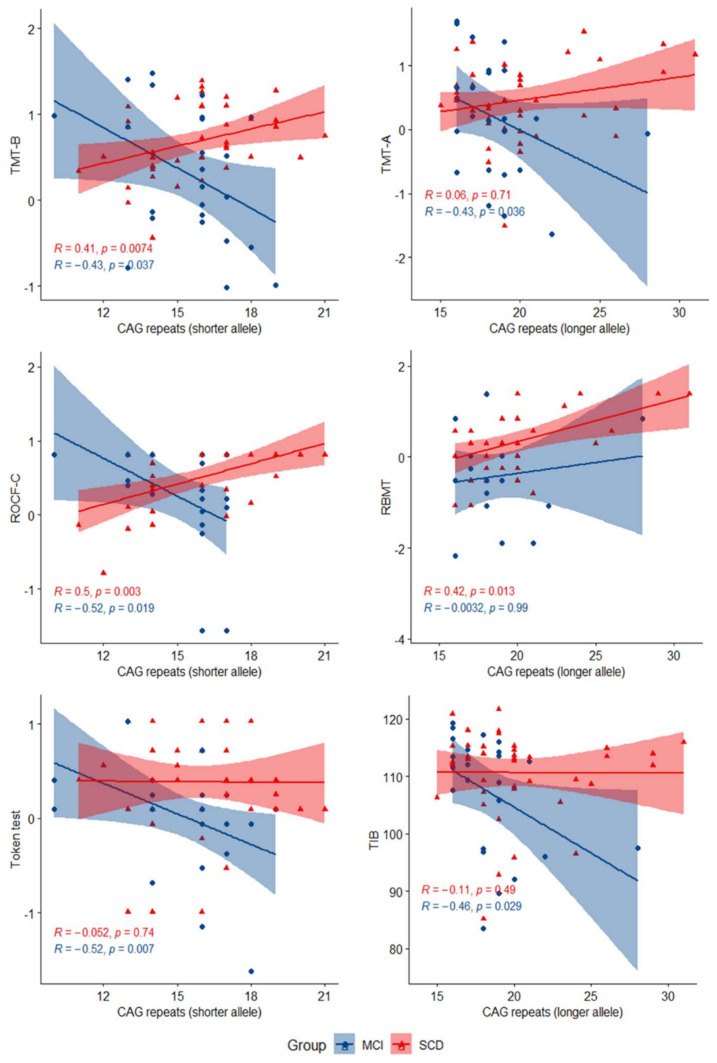
Scatter plots with lines of best fit (95% CI) showing the relationship between CAG repeat lengths and z-scores in neuropsychological tests in SCD and MCI. The Spearman correlation coefficient (R) and level of significance (p) are reported (statistical significance at *p* < 0.05).

In the SCD group, CAG repeat length in the longer allele was directly correlated with RMBT (R = 0.42, *p* = 0.013), while CAG repeat length in the shorter allele was significantly directly correlated with TMT-B (R = 0.41, *p* = 0.007) and ROCF-C (R = 0.50, *p* = 0.003). In the MCI group, CAG repeat number in the longer allele was inversely correlated with TMT-A (R = −0.43, *p* = 0.036) and TIB (R = −0.46, *p* = 0.029); CAG repeat length in the shorter allele was inversely correlated with TMT-B (R = −0.43, *p* = 0.037), ROCF-C (R = −0.52, *p* = 0.019), and the Token test (R = −0.52, *p* = 0.007) (Figure 2).

### 3.4. Multivariate Analysis and Regression Models

To ascertain that the associations between CAG repeat length and neuropsychological test scores were independent from confounding factors, we performed a multiple regression analysis. We considered CAG repeat length, age at baseline, sex, APOE and BDNF genotype, disease duration, and years of education as covariates. There was no evidence of multicollinearity among covariates, as assessed by tolerance values greater than 0.10. In the SCD group (Table 2), CAG repeat number remained significantly associated with TMT-B (B = 0.083 (95% CI = 0.032:0.134), *p* = 0.002), ROCF-C (B = 0.106 (95% CI = 0.027:0.184), *p* = 0.010), and RBMT (B = 0.082 (95% CI = 0.006:0.158), *p* = 0.036). In the MCI group, (Table 3) CAG repeat length remained inversely associated with ROCF-C (B = −0.177 (95% CI = −0.345: −0.009), *p* = 0.041) and TIB (B = −1.113 (95% CI = −1.855: −0.371), *p* = 0.006). Logarithmic models best described the relationship between CAG repeat length and TMT-B (R^2^ = 0.143, *p* = 0.015), ROCF-C (R^2^ = 0.315, *p* = 0.001), and RBMT (R^2^ = 0.222, *p* = 0.004) in the SCD group. In the MCI group, a linear regression for ROCF-C (R^2^ = 0.198, *p* = 0.049) and a logarithmic equation for TIB (R^2^ = 0.193, *p* = 0.036) best described the relationship between these tests and CAG repeats.

## 4. Discussion

The HTT gene is mainly known in its expanded variant as this mutation causes HD. Nevertheless, HTT CAG repeats below the disease threshold have been showed to play a vital role in evolution and intelligence [22]. More recently, CAG repeats in the intermediate range have been linked to depression [23,24,25], AD [26], and the non-fluent variant of primary progressive aphasia [27].

In this work, we focused on the effect of HTT CAG repeats and IAs on cognitive functions in a sample of patients who complaint about cognitive decline (SCD) or presented objective cognitive impairment (MCI). We showed that the number of CAG repeats in HTT gene influenced memory, visual–spatial ability, executive function, and language in these groups of patients. In particular, we found a direct correlation between CAG repeat length and neuropsychological scores in SCD patients but an inverse correlation in the MCI group. Furthermore, we found an inverse association between CAG repeats and premorbid intelligence measured by TIB in MCI patients. To the best of our knowledge, this has been the first study assessing for an effect of HTT CAG repeats on cognitive functions in SCD and MCI.

Lee et al. recently reported that the number of repeats in HTT confers advantageous changes in brain structure and that it was directly associated with general intelligence and visual–perceptual skills in a large cohort of children with a number of repeats below the disease threshold [22]. This favorable effect is also supported by neuroimaging data showing that a higher repeat number is related to increased gray matter within the pallidum in healthy subjects [40].

At the same time, Lee et al. showed that within the disease-causing range, the relationship is inverse: the greater the number of repeats, the worse the cognitive function. Therefore, the authors speculated that the effects of CAG repeats in HTT may have a non-linear, inverted U-shape relationship, with advantageous changes occurring below the disease threshold, and that once above disease threshold, increasing CAG repeats result in poorer cognitive function.

Our results, if confirmed by further studies on larger sample, may add another piece to the picture, suggesting that the dual effect of CAG repeats on cognitive functions may depend on the global cognitive status, as well as in subjects who are carriers of the wild-type HTT. In other words, a higher number of CAG repeats in the HTT gene might enhance cognitive function in cognitively healthy subjects. Nevertheless, as cognitive impairment advances, this advantage fails, and longer repeat length begins to play a detrimental effect.

A similar dimorphic effect has also been shown with cognitive reserve [41,42] and BDNF Val66Met polymorphism [43]. Stern’s model of cognitive reserve assumes that highly intelligent or educated individuals appear to be able to cope better with the presence of a neurodegenerative pathology, maintaining a normal functional level for a longer time than less educated people [41], but when this reserve is overcome, a faster rate of decline after the onset of clinical symptoms is evident [41,42].

In a previous work on the same sample of patients considered in the present analysis, we showed that Val66Met polymorphism of BDNF increased the risk of progression from SCD to MCI but, in patients who progressed to MCI, the wild-type BDNF gene was associated with a more rapid progression [43].

The interaction among HTT, BDNF, and cognitive reserve is an interesting topic. BDNF has been indicated as a candidate underlying mechanism of cognitive reserve, through neural plasticity [44]. Wild-type Huntingtin protein specifically enhances the vesicular transport of BDNF along microtubules [10]. Interestingly, we found that the higher the number of CAG repeats, the lower the premorbid intelligence (a cognitive reserve proxy) in MCI patients. We could speculate that, in MCI patients, a higher number of CAG repeats in the HTT gene negatively influences the interaction with BDNF and, therefore, with cognitive reserve.

Nevertheless, due to the size of our sample, we did not further explore this point. For instance, it would be interesting to reproduce the analysis in subsamples of patients grouped according to BDNF genotype as well as class of cognitive reserve. We aim to provide further information on this topic in future works in a larger sample.

The molecular mechanism by which increasing HTT repeats could translate into variability in function may be related to subtle changes in the way HTT creates multiprotein complex formations [19]. In Vitro studies have demonstrated that wild-type Huntingtin is neuroprotective in brain cells exposed to various apoptotic stimuli [45]. PolyQ tracts stabilize protein interactions [46], possibly optimizing the conformation and enhancing the function of the polypeptides. The function of the protein might be linked to the length of polyQ tracts through a non-linear relation with the best function reached at an intermediate number of CAG repeats and then showing a progressive decrease [22]. In line with this evidence, we showed that logarithmic regression models best explain the relations between CAG repeat length and neuropsychological scores, suggesting that the strength of the effect of CAG repeats on cognitive function tends to reduce as the number of repeats grows.

Regarding IA, we reported an almost high prevalence of IA in SCD and MCI patients, corresponding to the upper limit of confidence intervals described in previous studies on general populations [47,48,49] but consistent with a study by Sequeiros et al. [50]. As a control group was not available in our study, we cannot evaluate if IAs constitute a risk factor for development of SCD and MCI. This was the first limitation of our study. Another limitation of the study was the relatively small sample size. Due to this limitation, we could not compare psychometric variables between IA+ and IA- groups. Furthermore, as this was a single-center study, there may have been biases regarding assessment, diagnosis procedures, and inclusion of only Caucasian participants. The recruitment method should also be considered, as demographic and neuropsychological features are different according to the recruitment method [51]. In our work, memory clinic patients were included; therefore, our results might not be able to be extended to general population.

Finally, the lack of data about follow-up, cardiovascular risk factors, brain imaging, and AD biomarkers represents another limitation of our study. Considering these limitations, our results should be considered as preliminary. We aim to replicate these analyses on an independent larger sample, also including healthy controls, to confirm our findings. However, this work has some remarkable strength. First, we are not aware of previous works assessing for the effect of HTT gene variants on cognitive function in SCD and in MCI. Our results could contribute to understanding the mechanism underlying the progression from normal cognition to dementia. The inclusion of a wide number of demographic, cognitive, and genetic variables represents a strength of our work.

## 5. Conclusions

In conclusion, for the first time, we demonstrated that the HTT gene is involved in neuropsychological functions in individuals experiencing SCD or MCI. Moreover, we suggested that this effect depends on the cognitive status of subjects. Our results leave several open questions about how HTT interacts with other genetic factors and the cognitive reserve in influencing the progression of cognitive decline. Future studies including longitudinal data will be able to explore this issue, adding key information for our understanding of the cognitive process and of cognitive changes through the spectrum of AD.

## Figures and Tables

**Table 1:** Comparison of demographic, cognitive, and genetic features between SCD and MCI.

Variable	SCD	MCI	*p*
N	46	29	
Age at onset (years)	56.00 (15.00)	66.00 (13.00)	<0.001
Age at baseline (years)	61.52 (14.13)	68.53 (13.44)	0.001
Disease duration (years)	3.53 (2.84)	2.70 (2.91)	0.096
Sex (women/men)	34/12	19/10	0.437
Family history of dementia	52.17% (33.99–70.35)	55.17% (37.07–73.27)	0.800
Education (years)	11.00 (8.00)	8.00 (8.00)	0.004
*APOE* ε4^+^	28.26% (15.25–41.27)	27.59% (11.31–43.85)	0.949
CAG repeats, shorter allele	16.00 (3.00)	16.00 (2.50)	0.376
CAG repeats, longer allele	19.00 (3.25)	18.00 (2.50)	0.042
IA^+^	3/46	2/29	0.949
MMSE	27.15 (3.85)	26.70 (1.70)	0.094
HDRS	5.00 (6)	5.00 (6.00)	0.691
MAC-Q	26.00 (2.00)	25.00 (6.00)	0.401

Values quoted in the table are medians and interquartile ranges (IQR), frequencies, and percentages (95% CI). *p* indicates the level of significance for the comparison between SCD and MCI (statistical significance at the *p* < 0.05, in underlined characters). Abbreviations: MMSE = Mini-Mental State Examination; HDRS = Hamilton Depression rating scale; MAC-Q = Memory Assessment Clinics-Questionnaire.

**Table 2:** Multiple regression analysis for TMT-B, ROCF-C, and RBMT in the SCD group.

	TMT-B				ROCF-C				RBMT		
	B	95% CI			B	95% CI			B	95% CI	
		Lower	Upper			Lower	Upper			Lower	Upper
Constant	−1.624 *	−2.923	−0.324		−0.657	−2.276	0.962		−0.085	−2.809	2.638
CAG repeats (shorter allele)	0.075 *	0.013	0.137		0.098 **	0.027	0.169		0.019	−0.110	0.148
CAG repeats (longer allele)	0.010	−0.026	0.046		−0.009	−0.055	0.037		0.076 *	0.001	0.152
Age at baseline (years)	0.020 **	0.006	0.033		−0.001	−0.018	0.015		−0.022	−0.050	0.005
Disease duration (years)	0.011	−0.022	0.044		0.006	−0.033	0.045		0.018	−0.051	0.087
Education (years)	−0.028	−0.056	0.000		−0.020	−0.055	0.014		0.004	−0.050	0.058
Female sex	0.017	−0.247	0.280		0.051	−0.303	0.406		−0.293	−0.839	0.254
*APOE* ε4^+^	0.075	−0.171	0.321		−0.057	−0.378	0.265		−0.278	−0.797	0.240
*BDNF* Val66Met^+^	−0.065	−0.298	0.168		0.127	−0.172	0.426		0.247	−0.188	0.681

Unstandardized regression coefficient (B); 95% confidence intervals (95% CI) are reported. Abbreviations: TMT-B, ROCF-C, and RMBT were considered as dependent variables. There was no evidence of multicollinearity among the independent variables (tolerance values > 0.10). Abbreviations: TMT-B = Trail Making Test part B; ROCF-C = Rey–Osterrieth Complex Figure copy; RMBT= Rivermead Behavioral Memory Test. Statistical significance at *p* < 0.05: * *p* < 0.05, ** *p* < 0.01, *** *p* < 0.001.

**Table 3:** Multiple regression analysis for ROCF-C and TIB in the MCI group.

	ROCF-C				TIB		
	B	95% CI			B	95% CI	
		Lower	Upper			Lower	Upper
Constant	0.938	−2.951	4.827		117.372	97.847	136.897
CAG repeats (shorter allele)	−0.175 *	−0.342	−0.009		−1.081 **	−1.737	−0.426
CAG repeats (longer allele)	0.083	−0.032	0.198		0.244	−.208	0.696
Age at baseline (years)	−0.004	−0.056	0.049		−0.060	−0.291	0.170
Disease duration (years)	−0.003	−0.079	0.073		−0.219	−0.531	0.094
Education (years)	0.076	−0.018	0.170		1.803 ***	1.421	2.185
Female sex	−0.440	−1.179	0.299		−9.084 ***	−12.077	−6.091
*APOE* ε4^+^	−0.120	−0.871	0.631		6.141	3.322	8.959
*BDNF* Val66Met^+^	0.380	−0.374	1.135		0.541	−2.214	3.497

Unstandardized regression coefficient (B); 95% confidence intervals (95% CI) are reported. ROCF-C and TIB were considered as dependent variables. There was no evidence of multicollinearity among the independent variables (tolerance values > 0.10). Abbreviations: ROCF-C = Rey–Osterrieth Complex Figure copy; TIB = Brief Intelligence Test. Statistical significance at the *p* < 0.05: * *p* < 0.05, ** *p* < 0.01, *** *p* < 0.001.

## Data Availability

Data that support the findings of this study will be shared upon request from any qualified investigator.
